# Whose data can we trust: How meta-predictions can be used to uncover credible respondents in survey data

**DOI:** 10.1371/journal.pone.0225432

**Published:** 2019-12-02

**Authors:** Sonja Radas, Drazen Prelec

**Affiliations:** 1 Institute of Economics, Zagreb, Croatia; 2 Massachusetts Institute of Technology, Cambridge, Massachusetts, United States of America; Shandong University of Science and Technology, CHINA

## Abstract

Many areas of economics use subjective data, although it had been known to present problems regarding its reliability. To improve data quality, researchers may use scoring rules that reward respondents so that it is most profitable for them to tell the truth. However, if the subjects are not well informed about the topic or if they do not pay sufficient attention, they will produce data that could not be dependably used for decision-making even though subjects gave their honest answer. In this paper we show how meta-predictions (respondents’ predictions about choices of others) can be used for identification of respondents who produce dependable data. We use purchase intention survey, a popular method to elicit early adoption forecasts for a new concept, as a test bed for our approach. We present results from three online experiments, demonstrating that corrected purchase intentions are closer to the real outcomes.

## Introduction

Many areas of economics use subjective data, gathered from respondents via traditional surveys as well as via new web-based information exchanges and markets. At the same time, there has always been concern about the quality and reliability of such data. To motivate honest and careful answers, researchers can use scoring rules that provide financial or reputational incentives [1, 2]. However, even honest respondents may not be well informed about the topic or they may not pay sufficient attention to the task. Since during data collection it is usually not possible to differentiate “bad” respondents from the “good” ones, pooling responses from both groups can easily produce unreliable data.

In this paper we propose a method for identifying “bad” respondents whose data is not dependable, and we show that removing the information gathered from them improves the quality of collected data. As the test bed we use purchase intentions survey, a method frequently used in new product development. Purchase intentions are used to predict future sales and are collected throughout the product development via easily administered questionnaire that asks respondents if they want to buy a specific product at a specific price. As purchase intentions surveys are usually conducted in early phases of product development, respondents are shown only a product concept or an early prototype. Therefore they need a certain level of product and category understanding to produce dependable answers. Aside from that, some respondents may give untrustworthy answers because they simply do not understand the questionnaire or they do not pay sufficient attention during the survey. Obviously, mixing information from the non-credible group with the information from the credible one will negatively affect the accuracy of any subsequent inferences and predictions. We propose to identify and discard the information from the lower-quality respondents, so that inferences can be made based on the information collected from the high-quality respondents only.

We develop a theoretical model of how credible and non-credible respondents make purchase intention predictions, resulting in an easy to follow algorithm to identify both groups. The theoretical model is based on the combination of the classic purchase intention question (“would you buy”) and meta-prediction that is assessed by asking which percentage of others would buy. Our theoretical model uses this meta-prediction feature to identify people who produce dependable answers. We show that credible respondents are in fact those people who are the best in predicting their peers’ intentions.

We illustrate the method on data collected in three online experiments, where we compare the purchase intentions with real outcomes. In the first experiment the outcomes are represented by the choices of respondents other than those who provided purchase intentions. In the other two experiments, outcomes are measured by real purchases of the same respondents who provided intentions. Results show that the purchase intentions adjusted by our algorithm approximate the real purchases better than the traditional ones.

### Literature review

Here we give a short presentation of the relevant literature.

#### Purchase intentions literature

Eliciting purchase intention is a very popular technique to estimate future sales, and as such is widely used in new product development. It is applied starting from early phases of product development, while product is still in the concept stage. Purchase intention survey is administered in the following way: first respondents are shown a product concept, which can be a product sketch, photograph, written description, a short video, or any combination of those. In later stages of product development they may be shown or given an actual prototype. After the respondents have been presented with the concept, they are asked if they would be willing to buy this product at a specific price. As purchase intentions are used to forecast the demand, traditionally the main research focus here is on ability to use intention data to accurately predict actual purchase incidence. Consequently, a lot of attention has been devoted to examining various factors that may affect the accuracy of purchase intentions and the ability to exactly forecast the demand.

Explanations for what factors affect predictive ability range from respondents’ systematic biases [3, 4], to the effects of time passage between when purchase intention survey is administered and when actual purchasing decision is made [5 6]. To improve the ability to forecast behavior from intentions, researchers have tested alternative scales [7, 8]. In addition, models were developed that account for biases in the measurement and reporting of intentions, the heterogeneity across customers, changes in true intentions between the time of the survey and the time of the purchase, and the stochastic and nonlinear nature of the relationship between intentions and behavior [9, 10, 11, 5, 6, 12, 13 14, 15]. In order to improve forecasts, Morwitz and Schmittlein in [16] segmented respondents according to demographic information and developed separate forecasts for each segment. To unify most of these factors, Sun and Morwitz in [17] proposed a model that incorporated “systematic intention biases, changes in true intentions over time, and the imperfect correlation between true intentions and actual purchasing”. Practically, such empirical models require large amount of data for estimation, in particular the data on past introductions of similar products where first the purchase intentions of consumers are measured and then their actual purchases are tracked. This amount and type of data is hard to come by, which is why this type of forecasting is usually performed by large consulting companies (such as Nielsen).

All the above approaches propose to adjust the intention scores by correcting for various factors, but they all make the same assumption that the people taking part in the survey understand the task, the questions, and the product, i.e. that they produce reliable data. Although potential subjects are often screened on their interest in product category, that does not necessarily mean that they really understand the concept, especially when it involves more than an incremental innovation. In this paper we propose to improve the reliability of data by retaining only the answers from “good” respondents, i.e. those who have necessary understanding and are paying attention. Our method aims to enhance the quality of collected data before any of the above models are applied, thus offering additional improvement to purchase intentions forecasting that is complementary to the existing models. Since we propose to increase data reliability, application of econometric models to reliable data will achieve better accuracy of predictions.

#### Relevant meta-predictions literature

Meta-predictions are respondents’ predictions of what others will do, and as such contain valuable information. Research in psychology has recognized that predictions about what others will do are more accurate predictors of individuals’ real behavior than their self-predictions [18]. Consistently with that, in political voting arena meta-predictions have been shown to produce better accuracy of election forecasts, as shown in [19] and [20]. In another setting, in [21] authors use “guess of guesses” where respondents guess the box office and are rewarded for how close their guess comes to the median of all guesses. Other research uses predictions of predictions to improve on traditional wisdom of the crowds [22, 23].

Meta-predictions can be used alone, or they can be combined with personal choice reporting to produce a mechanism like Bayesian Truth Serum or BTS [1], a game-theoretic scoring system that provides incentives for honest reporting of private judgments. BTS has been used successfully in situations where the ground truth is known, as well as in those where it is not known [1, 24].

The value of information contained in meta-predictions depends on their accuracy. In this paper we follow [1] that defines a prediction score that measures the quality of meta-predictions. We use this particular score because it is very natural, as it measures relative entropy between predictions and realizations. In addition, it can be used as a reward mechanism that incentivizes truthful responding.

## Methods

### Meta-predictions: The setup

In this section we start with a general game-theoretic setup, and continue with the description of the survey setting.

Let (*t*_1_, *t*_2_, …, *t*_*m*_) be player types and Ω = {*ω*_1_, *ω*_2_, …, *ω*_*n*_} states of the world. We assume that players are independent and identically distributed conditionally on the state of the world. We also assume that players have tacit knowledge about their peers and about the states of the world that allows them to make updates in a Bayesian fashion based on their signal. They all have a common prior *P*(*ω*_*i*_) over states of the world. All posterior beliefs about the states of the world, *P*(*ω*_*i*_|*t*_*j*_) are assumed to be consistent with Bayesian updating over the prior *P*(*ω*_*i*_). Assuming two different players, i.e. assuming that *r* ≠ *s*, player *r* can compute the expected of his posterior probability which gives the probability that player *s* is of type k or *P*(player *s* is of type *t*_*k*_|player *r* is of type *t*_*j*_). Players assume that people of the same type have the same expectations of type frequencies, and, conversely that different types have different expectations. This is effectively equating the whole type with a generic respondent (mathematically this is a consequence of the assumed Ω − i.i.d. condition for players). For this reason the above expectations could be written as *P*(*t*_*k*_|*t*_*j*_).

Next we explain how the above game-theoretical framework looks in survey setting. In a survey, different signals (and consequently different respondent types) are identical with chosen answers to multiple choice questions. Here we follow the setup in [1] and revisit the most relevant features of that exposition.

Assume that we have a particular question which has a finite number of possible alternative answers, say (*t*_1_, *t*_2_, …, *t*_*m*_). In survey setting these *m* answers correspond to *m* different player types. Respondents are asked: (1) to choose one and only one answer to the question (i.e. to declare their type), and (2) to give their estimate of the percentage of other respondents who chose any of the *m* alternative answers (i.e. to give their meta-predictions). For a particular respondent *i*, from (1) we obtain the vector of answers $\left(x_{1}^{i},x_{2}^{i},\ldots ,x_{m}^{i}\right)$, where $\sum_{j=1}^{m}x_{j}^{i}=1$, and all the coordinates except the one corresponding to the chosen answer/type are zero. From (2) we obtain the vector $\left(y_{1}^{i},y_{2}^{i},\ldots ,y_{m}^{i}\right)$, where $y_{j}^{i}$ is the estimated percentage of respondents who choose answer j as predicted by the respondent *i*. In other words, while (1) yields respondent’s own type, (2) gives her estimates of the overall distribution of all answers/types.

We define two more variables. By $\overset{-}{x_{j}}$ we denote the average $\overset{-}{x_{j}}={lim}_{n\to \infty }\frac{1}{n}\sum_{r=1}^{n}x_{j}$. This is actually the frequency of the chosen answer *j* in the sample. For this particular answer, by $\overset{-}{y_{j}}$ we denote the geometric mean of all player predictions $y_{j}^{i}$, where *i* goes over all the respondents. In other words, $\bar{y_{j}}=\prod_{i=1}^{\infty }y_{j}^{i}$, or $log\left(\bar{y_{j}}\right)={lim}_{n\to \infty }\frac{1}{n}\sum_{i=1}^{n}log(y_{j}^{i})$. Now we define the prediction score PS for a respondent *i* as defined in [1]:

$${PS}^{i}=\sum_{j=1}^{m}\overset{-}{x_{j}}log\frac{y_{j}^{i}}{\overset{-}{x_{j}}}$$ (1)

Note that the prediction score is optimized when the prediction exactly matches the actual frequencies of the answer (and in that case, the score is zero). The score measures relative entropy between predictions and realizations, and it is also a truth inducing scoring rule by itself, as shown in [1]. (In applications we can encounter extreme predictions such as 0% and 100%. In order to be able to compute predictions scores we change those to 0.1% and 99.9%).

### Theoretical model

We start with a theoretical model to produce insights about how to find credible respondents, which we later validate in a survey. As stated earlier, our key assumption is that there are two groups of people in the population. Some people are “low-quality” respondents whose answers are undependable, because they do not understand the product or context, cannot relate to the questions, do not pay sufficient attention, or any combination of the above. The others are “high-quality” respondents who provide dependable answers, because they understand the task and context, are able to accurately judge the product, and they pay attention to the survey.

#### Model setup

First we define states of the world and respondent types. States of the world are identical to the product quality: as the product can be either good or bad, we can be in a high quality state *ω*^*h*^ or a low quality state *ω*^*l*^. These states of the world are probabilistic, where *P*(*ω*^*h*^) = *p*. Researcher does not know which state of the world has been realized.

We define the respondent types in the following way. Credible respondents pay attention and understand the product, and are able to tell whether the product is of high or low quality. In contrast, the non-credible respondents cannot discern whether the product is good or bad, and thus they basically guess the state of the world. Both credible and non-credible respondents can choose to buy or not depending on other factors that we do not explicitly model here. All respondents of the same type are homogeneous: they all give the same responses and meta-predictions.

Therefore, in the high quality state of the world *ω*^*h*^ we have the following respondent types:

*T*_1_ = credible respondents who know the quality is high and want to buy,
*T*_2_ = credible respondents who know the quality is high but do not want to buy,
*T*_5_ = non- credible respondents who want to buy,
*T*_6_ = non- credible respondents who do not want to buy.

Similarly, in the low quality state of the world *ω*^*l*^ we have:

*T*_3_ = credible respondents who know the quality is low and want to buy,
*T*_4_ = credible respondents who know the quality is low but do not want to buy,
*T*_5_ = non- credible respondents who want to buy,
*T*_6_ = non- credible respondents who do not want to buy.

We start by defining the matrix Q of joint probabilities P(ω_i_ ∩ T_j_) for the chosen respondent category and the state of the world (here ω_i_ is used as a generic label for any of the two states {ω^h^, ω^l^}). We assume that respondents know the joint probability matrix of respondent types and states of the world Q. This information is private and inaccessible to the researcher who is able to distinguish only between those respondents who answer Yes and those who answer No.

*Q* is a 6 x 2 matrix where the first four rows represent credible respondents, the last two rows represent non- credible respondents, and columns represent the states of the world. The probability of a respondent being credible is denoted by *λ*. The matrix *Q* is presented in Table 1.

**Table 1:** Matrix *Q* of joint probabilities.

	*ω*^*h*^	*ω*^*l*^
*T*_1_	*λap*	0
*T*_2_	*λ*(1 − *a*)*p*	0
*T*_3_	0	*λc*(1 − *p*)
*T*_4_	0	*λ*(1 − *c*)(1 − *p*)
*T*_5_	(1 − *λ*)*δp*	(1 − *λ*)*δ*(1 − *p*)
*T*_6_	(1 − *λ*)(1 − *δ*)*p*	(1 − *λ*)(1 − *δ*)(1 − *p*)

Here *a* is the probability that credible people will buy in the state *ω*^*h*^, and *c* is the probability that credible people will buy in the state *ω*^*l*^. The non- credible respondents are not certain which state of the world they are in (as they can not tell the product quality), so they express positive intentions with probability *δ* in both states. Notice that *a* > *δ*, since for the same level of demand, people who know that the product is good will be more likely to buy than those who are uncertain about the quality. Similarly, people who know that the product is bad will exhibit smaller probability to purchase, which means that *δ* > *c*. The associated matrix of posteriors QP is presented in Table 2. (matrix QP contains posterior probabilities $P(\omega_{i}|T_{j})=\frac{P(\omega_{i}\cap T_{j})}{P(T_{j})}$, *ω*_*i*_ ∈ {*ω*^*h*^, *ω*^*l*^}).

**Table 2:** Matrix *QP* of posteriors.

	*ω*^*h*^	*ω*^*l*^
*T*_1_	1	0
*T*_2_	1	0
*T*_3_	0	1
*T*_4_	0	1
*T*_5_	*p*	1 − *p*
*T*_6_	*p*	1 − *p*

Notice that respondents *T*_1_, *T*_2_ “live” only in the high quality state of the world, while *T*_3_, *T*_4_ “live” only in the low quality state. Percentage of positive purchase intentions (i.e. probability of answer *Yes*) is obtained by adding the contributions from all respondent types who answer *Yes*. More precisely, in high quality states *ω*^*h*^ that probability, denoted by *α*, comes from respondents *T*_1_ and *T*_5_, and is equal to *λa* + (1 − *λ*)*δ* = *α*, while probability of the answer *Yes* in low quality states *ω*^*l*^, denoted by *β*, is equal to *λc* + (1 − *λ*)*δ* = *β* and comes from types *T*_3_ and *T*_5_. It is easy to see that *a* > *δ* implies *a* > *α*. Similarly, *δ* > *c* implies *β* > *c*.

#### Meta-predictions

It is important to note that all the tacit information that respondents have and that allows them to engage in Bayesian updating is unobservable. The researcher who conducts the survey observes only *Yes-No* answers and respondents’ peer predictions for *Yes-No* answers. In order to link the private knowledge with the information that is observable, we need to understand how respondents use their private knowledge to make predictions.

First, our respondents can use Bayes rule and their private information from matrices Q and QP to compute conditional probabilities of belonging to a chosen type. These are *P*(*T*_*k*_|*T*_*j*_), and they can be calculated with the help of the formula

$$P(T_{k}|T_{j})=\sum_{\omega_{i}}\frac{P(\omega_{i}|T_{k})P(\omega_{i}|T_{j})P(T_{k})}{P(\omega_{i})}$$ (2)

If the respondent of the type *T*_*j*_ is credible, she knows which quality level has been realized, and therefore she knows if she is in high quality state of nature or low quality state of nature. She knows that in the high quality state positive purchase intentions come from respondents *T*_1_ and *T*_5_, while in the low quality state they come from respondents *T*_3_ and *T*_5_. She can use this information and meta-predictions *P*(*T*_*k*_|*T*_*j*_) from the complete information structure to calculate *P*(*Y*|*T*_*j*_). More precisely:

$$P(Y|T_{1})=P(T_{1}|T_{1})+P(T_{5}|T_{1}),P(Y|T_{2})=P(T_{1}|T_{2})+P(T_{5}|T_{2})$$

in *ω*^*h*^, and

$$P(Y|T_{3})=P(T_{3}|T_{3})+P(T_{5}|T_{3}),P(Y|T_{4})=P(T_{3}|T_{4})+P(T_{5}|T_{4})$$

in *ω*^*l*^.

Contrary to that, the unreliable respondents are not sure which level of quality has been actualized, so they are not certain about the state of the world they are in. Therefore they include respondents from both high and low quality states of the world when they calculate meta-predictions. This is why unreliable respondents always do the same calculation, and that is

$$P(Y|T_{5})=P(T_{1}|T_{5})+P(T_{3}|T_{5})+P(T_{5}|T_{5}),$$

for the respondent type *T*_5_, and

$$P(Y|T_{6})=P(T_{1}|T_{6})+P(T_{3}|T_{6})+P(T_{5}|T_{6}),$$

for the respondent type *T*_6_.

It is easy to show that in *ω*^*h*^, *P*(*Y*|*T*_1_) = *P*(*Y*|*T*_2_) = *α*, while in *ω*^*l*^, *P*(*Y*|*T*_3_) = *P*(*Y*|*T*_4_) = *β*. In other words, in both states of the world the reliable respondents perfectly predict the realized choices of their peers. This follows from the assumption that the choice distribution is a function of quality, and reliable respondents know quality. Since ignorant respondents do not recognize quality, their meta-predictions are the same in both states of the world, and they are equal to *P*(*Y*|*T*_5_) = *P*(*Y*|*T*_6_) = *pα* + (1 − *p*)*β*. These predictions are between *α* and *β*, which means that in both states of the world peer predictions of types *T*_5_ and *T*_6_ are inaccurate.

### Identifying credible respondents

As all the information involving respondent types is private knowledge, the outcomes of the purchase intention questionnaire are only *Yes* and *No* answers and respondents’ meta-predictions of these answers. In other words, from the survey administrator’s point of view, there are only two types of players, those who say *Yes* and those who say *No*. These personal answers, realized shares of votes $\overset{-}{x_{Y}}=P(Y|\omega^{i}),\overset{-}{{\;x}_{N}}=P(N|\omega^{i})$, and peer predictions $y_{Y}^{T_{i}}=P(Y|T_{i})$ are used to compute prediction scores ${PS}^{T_{i}}=\overset{-}{x_{Y}}log\frac{y_{Y}^{T_{i}}}{\overset{-}{x_{Y}}}+\;\overset{-}{x_{N}}log\frac{y_{N}^{T_{i}}}{\overset{-}{x_{N}}}$ in the *Yes-No* context. Notice that in practice when the score is applied to surveys it is admissible that members of the same type differ in their peer-predictions. For example, in the state *ω*^*h*^ we can have that *P*(*Y*|*T*_1_) ≠ *P*(*Y*|*T*_5_) for different subtypes *T*_1_ and *T*_5_ within the type *Yes*.

Computing the prediction scores in the *Yes-No* context, we find that the credible respondents have higher scores regardless of their personal answer, which follows from our assumption that they predict perfectly. This is stated in Proposition 1.

***Proposition 1***.

*In both states of the world the respondents achieving highest prediction scores for Yes-No answers are identical with the credible respondents*.

Proof: By assumption, credible respondents know the state of the world, and hence also the distribution of types. Therefore they will receive the optimal prediction score (zero).

Proposition 1 implies that it is possible to identify credible and non- credible respondents by computing prediction scores. Once the non-credible respondents are recognized, we can discard the information provided by them and retain only the data that was collected from the credible people. In other words, instead of settling for purchase intention estimate being the ratio of all buyers over all respondents, we produce corrected estimate by using the answers from credible respondents only. This corrected estimate *k* is computed as the ratio of credible buyers in the set of all reliable individuals:

$$k=\frac{credible\;buyers}{all\;credible\;responents}$$

By using this corrected purchase intention rate *k* we eliminate the “noise” coming from non-credible respondents. If in addition we can use information score to establish which state of the world has been realized, as shown in the Proposition 2.

The Proposition 1 allow us to state an easy algorithm for practical use in purchase intention surveys. The algorithm consists of four easy steps as follows.

**Algorithm**
- Step 1: Computation of predictions scores and information scores (which form the BTS scores).
- Step 2: Identification of the respondents with the highest prediction scores: they are the credible ones.
- Step 3: Computation of the corrected purchase intention rate *k*.

#### Generalizations

Our approach can be generalized to situations where there are more than two possible options. In practice, purchase intentions can be elicited by offering subjects several positive and negative options (for example “definitely will buy” “likely will buy”, “likely will not buy”, and “definitely will not buy”). We show that regardless of the number of offered answers, credible respondents are always those who have the highest prediction scores. Algorithm from above can still be used, with small modifications to accommodate larger number of options. Detailed discussion is presented in the S1 Appendix.

In this paper we used purchase intention survey as the test bed for our approach. Any survey related to choice can be approached in the same way, as long as the two states of the world are related to the respondents’ relevant expertise. In other words, the crucial assumption to be satisfied is that those people who have the knowledge are able to discern which state of the world has been realized. For example, voting for a political candidate could be seen in this setting, with some respondents being able to correctly assess the candidate’s overall quality (the two states of the world being “this is a good candidate” and “this is a bad candidate”).

Further generalization comes in increasing the number of states of the world. One can argue that there are many nuances in quality (as in everything else), and that specifying just two outcomes is overly limiting. We show that one can indeed assume as many states of the world as desired: again the credible respondents in any of those states are identical to the respondents with the highest prediction scores (proof in the S1 Appendix).

## Experimental results

We illustrate the use of the method with the aide of three purchase intention experiments. Products that were chosen for experiments were very new at that time, and as such they required a certain amount of knowledge to be properly understood. The products were described by a short text (one or two paragraphs long) listing the most important features, and by two or three photographs. To insure that these products are new and non-trivial, we perused the crowd-funding platforms like IndieGoGo and KickStarter. We were looking for products that were funded through those platforms, only recently became available commercially, and were relatively affordable. These characteristics ensured that respondents needed to understand the product and category, and also had to pay attention to the survey. Very basic descriptions of the products are presented in Table 3.

**Table 3:** Product descriptions.

Product 1	A device that uses smart phone to produce holographic images
Product 2	Foldable flower planter from biodegradable material
Product 3	Charger that allows for simultaneous charging of several electronic devices
Product 4	A small Bluetooth tracker
Product 5	A small perforated board for organizing/keeping small objects
Product 6	A metal USB drive
Product 7	An advanced wireless lighting system in a light bulb
Product 8	A product which turns iPhone and iPad into a smart universal remote
Product 9	Car ionizer that cleans air by producing a stream of negative ions
Product 10	Bluetooth smart watch

All experimental work was performed with the online subject panel MTurk. Experiments were approved by the Massachusetts Institute of Technology Committee on the Use of Humans as Experimental Subjects (Protocol number: 1802252061). At the beginning of the questionnaire, respondents were presented with the text informing them about the nature of the experiment and the payment, which included a bonus for the best predictors. Afterwards the respondents were asked to press a button if they were 18 years of age and consented to the experimental conditions. All respondents who accessed the questionnaire link also gave their consent.

In experiments we compared the corrected and traditional purchase intentions with the real outcomes. In the first experiment we used one group of respondents to provide intentions and a different group to provide measures of purchase incidence. In contrast, in the second and third experiment we used purchase incidence by the same group that provided intentions.

We considered each one of the product-price combinations separately. For a given product-price combination we asked two questions: “will you buy” and “what percentage of your peers will buy”. We should point out that our insights were derived from a theoretical model, but in practical applications we cannot expect that respondent types are homogeneous, i.e. we cannot assume that all the people of the same type give exactly the same answers. However, we can assume that the answers from people who belong to the same type belong to the same distribution.

For a given product-price combination, following the algorithm from section 5, we first computed prediction scores (step 1) and rank-ordered them from the highest to the lowest (step 2). By Proposition 1 we know that credible respondents are those with the highest prediction scores. We assumed that participants belonged to two segments that corresponded to two different distributions (credible respondents, and non-credible respondents), and then we conducted segmentation of the prediction scores by applying finite mixture models (FMM). Finite mixture models have been used before in latent segmentation [25,26, 27]. Assuming two classes, we estimated the following simple model:

$$f(y)=\sum_{i=1}^{2}\pi_{i}f_{i}(y),$$ (3)

where variable *y* contains prediction scores, *π*_*i*_ is the probability for the *i*-th class (i.e. segment), 0 ≤ *π*_*i*_ ≤ 1, $\sum_{i=1}^{2}\pi_{i}=1$, and *f*_*i*_(.) is the conditional probability density function for the observed response in the *i*-th class model. As candidates for distributions *f*_*i*_(.) we used lognormal family, exponential family, and normal family: this means that every segmentation was carried out by three models that we present in parallel in Tables 4 and 5.

**Table 4:** Corrected rates of purchase intention rates and corresponding errors in Experiment 1—Real purchasing decision is the decision to enter the lottery.

					Normal mixture		Lognormal mixture		Exponential mixture	
Product	Price in $	PINT Purchase intention	PINC Purchase incidence	Error*	Correct. rate k	Correct. error**	Correct. rate k	Correct. error**	Correct. rate k	Correct. error**
P1	5	0.62	0.39	0.23	0.62	0.23	0.62	0.23	0.62	0.23
	7	0.44	0.19	0.25	0.34	0.15	n/a	n/a	0.37	0.18
	9	0.29	0.11	0.18	0.1	0.01	0	0.11	0.1	0.01
P2	5	0.41	0.26	0.15	0.22	0.04	0.42	0.16	0.22	0.04
	7	0.22	0.1	0.12	0.09	0.01	0.17	0.07	0.08	0.02
	9	0.15	0.06	0.09	0.02	0.04	0.01	0.05	0.02	0.04
P3	7	0.67	0.55	0.12	0.65	0.10	0.62	0.07	0.64	0.09
	11	0.54	0.36	0.18	0.49	0.13	0.48	0.12	0.51	0.15
	15	0.32	0.24	0.08	0.28	0.04	0.24	0.00	0.28	0.04
P4	6	0.82	0.71	0.11	0.91	0.20	0.77	0.06	0.9	0.19
	10	0.65	0.51	0.13	0.75	0.24	0.7	0.19	0.7	0.19
	14	0.33	0.30	0.03	0.17	0.13	0.29	0.01	0.2	0.10
P5	6	0.51	0.35	0.16	0.44	0.09	0.36	0.01	0.46	0.11
	10	0.16	0.16	0.00	0.15	0.01	0.08	0.08	0	0.16
	14	0.05	0.06	0.01	0.01	0.05	0.05	0.01	0.03	0.03
P6	4	0.74	0.60	0.14	0.74	0.14	0.71	0.11	0.74	0.14
	7	0.51	0.39	0.12	0.47	0.08	0.51	0.12	0.45	0.06
	10	0.25	0.22	0.03	0.15	0.07	0.08	0.14	0.14	0.07
P7	10	0.81	0.62	0.19	0.86	0.24	0.87	0.24	0.87	0.25
	15	0.50	0.39	0.11	0.49	0.10	0.48	0.09	0.50	0.11
	20	0.25	0.13	0.12	0.24	0.10	0.22	0.09	0.19	0.06
P8	10	0.40	0.38	0.03	0.37	0.01	0.12	0.25	0	0.38
	15	0.29	0.26	0.03	0.14	0.11	0.16	0.10	0.12	0.14
	20	0.17	0.12	0.04	0.05	0.07	0.12	0.00	0.05	0.07
P9	8	0.65	0.49	0.17	0.71	0.22	n/a	n/a	0.70	0.21
	11	0.48	0.22	0.26	0.43	0.21	0.44	0.22	0.42	0.20
	14	0.24	0.20	0.04	0.15	0.05	0.09	0.11	0.15	0.05
One-tailed Wilcoxon test: error vs. corrected error						0.26		0.32		0.48
One-tailed matched-pair t-test: error vs. corrected error						0.25		0.44		0.65

* Error is computed as the absolute difference between purchase intentions and real purchases

** Corrected error is computed as the absolute difference between the corrected purchase intention rate *k* and real purchases

n/a denotes cells where the maximization algorithm in fmm package did not converge, so it was not possible to compute corrected intention rates

**Table 5:** Corrected purchase intention rates and corresponding errors in Experiments 2 and 3.

				Normal mixture		Lognormal mixture		Exponential mixture	
	PINT Purchase intentions	PINC Purchase incidence	Error*	Correct. rate k	Correct. error**	Correct. rate k	Correct. error**	Correct. rate k	Correct. error**
Experiment 2–100 subjects per group									
Product 1	0.42	0.06	0.36	0.39	0.33	0.14	0.08	0.39	0.33
Product 2	0.22	0.01	0.21	0.2	0.19	0.17	0.16	0.08	0.07
Product 4	0.52	0.08	0.44	0.49	0.41	0.46	0.38	0.5	0.42
Product 5	0.28	0.05	0.23	0.15	0.1	0.13	0.08	0.14	0.09
Product 6	0.36	0.04	0.32	0.18	0.14	0.2	0.16	0.12	0.08
Product 10	0.58	0.11	0.47	0.51	0.4	0.5	0.39	0.5	0.39
Experiment 3–250 subjects per group									
Product 1	0.4	0.03	0.37	0.24	0.21	0.40	0.37	0.24	0.21
Product 4	0.44	0.05	0.39	0.34	0.29	0.30	0.25	0.38	0.33
Product 5	0.34	0.02	0.32	0.24	0.22	0.2	0.18	0.29	0.27
Product 6	0.44	0.02	0.42	0.34	0.32	0.28	0.26	0.28	0.26
Product 6 second time	0.43	0.02	0.41	0.38	0.36	0.40	0.38	0.37	0.35
Product 10	0.6	0.09	0.51	0.57	0.48	0.64	0.55	0.57	0.48
Wilcoxon one tailed test: error vs. corrected error					0.0002		0.0015		0.0002
One-tailed matched-pair t-test: error vs. corrected error					0.0001		0.0011		0.0002

* Error is computed as the absolute difference between purchase intentions and real purchases.

** Corrected error is computed as the absolute difference between the corrected purchase intention rate *k* and real purchases.

Remark: in a very few cases when prediction score is 0, (this happens only for perfect predictors) we substitute it by 0.0001 so that we can perform lognormal and exponential fmm without eliminating data from those respondents.

After segmentation was completed, the next step was to determine the corrected purchase intention rate. Every subject was assigned a probability of belonging to each of the two segments, which was determined through finite mixture model. Since the prediction scores were rank-ordered, the segment that contains people with the highest prediction scores can be assumed to be the segment that contains credible people (denoted as segment R), while the segment that contains those with the lowest prediction scores is the one populated with non-credible people (this segment is denoted by U). Therefore the probability of belonging to R can be interpreted as the probability of being a credible respondent, and the probability of belonging to U as the probability of being an non-credible respondent. For the respondent *j* we denoted the probability to be credible as *p*^*j*^(*R*). Next we define the variable *b*^*j*^ = *Int*^*j*^ ∙ *p*^*j*^(*R*), where *Int*^*j*^ = 1 if the respondent *j* expressed intention to purchase, and 0 otherwise. In other words, *b*^*j*^ is the variable that contains probability of being a credible respondent for potential buyers only. Next we compute the corrected purchase intention rate as

$$k=\frac{\sum_{i=1}^{N}b^{j}}{\sum_{i=1}^{N}p^{j}(R)}$$

where *N* is the number of all respondents. In this way, we divide the probability that buyers are credible by the total probability of being credible.

### Experiment 1

The number of subjects was 166 persons divided into two groups (one group consisted of 82 respondents, the other one of 84 respondents). Among other things, each group was shown images and descriptions of the first nine products (products 1–9 in Table 3). The order of the products was randomized. The products were offered at three price levels, where the highest one was at most 50% of the real price, and the smallest one was about 25% to 30% of the real price. The range for the lowest price was $4 to $7, and for the highest price it was $9 to $15. The lowest price was presented first, followed by the medium price, and finally the highest price. Each product was seen by one group as available for purchase, and by the other group as unavailable. In this way for each product we could compare the purchase intentions from one group with the actual decisions from the other group; the latter was used as a proxy for market outcome. The actual purchase decision in this experiment involved agreeing to enter a lottery and committing to buy a product if chosen. The answers from the respondents who saw the product as unavailable were used to collect purchase intention measures, while the decision to enter the lottery from those who saw the product as available served as a proxy for real purchases.

We considered each one of the 27 product-price combinations separately, produced ordered prediction scores and used finite mixture model segmentation as outlined in (3). We corrected purchase intentions using the sum of normal, lognormal and exponential distributions. The corrected purchase intention rate *k* for each distribution choice is presented in Table 4.

We can observe that the corrected purchase intentions *k* are rather robust across the three possible distribution types.

In order to examine prediction quality, we consider absolute values of the errors in Table 4 and use matched pairs analysis. As we expected corrected purchase intentions to produce smaller errors, we use the Wilcoxon one-tailed test and one-tailed matched-pair t-test (presented in Table 4). The results show that the absolute errors produced by the corrected purchase intention rate are on average somewhat smaller for normal and lognormal model (average of 0.11 for normal and lognormal model, compared to 0.12 when no correction is applied), but are not significantly different.

One possible reason for the lack of significance is that lottery is not a good proxy for real purchases. We used lottery because it was administratively easier to do with an online panel compared to engaging in multiple real purchasing transactions. Because of such setup, our respondents were most likely more optimistic in expressing their readiness to purchase (i.e. to enter the lottery with small probability to win) than they would have been if they had been faced with the certainty of making a transaction.

For that reason in the next two experiments we decided to measure purchase incidence by real purchases.

### Experiment 2

In this experiment we offered products for sale directly instead of selling them through a lottery. The experiment involved two groups of 100 people, where each group saw three products. Since the experiment required that we stocked the products that were offered for sale, we decided to drop the products that were too expensive or too bulky (product 3, product 7, product 8, and product 9). We also added product 10 from Table 3. First group of 100 respondents saw products 1, 2, and 4; the other group saw the products 5, 6, and 10. For each of the products we asked for subjects’ purchase intention; later in the survey we offered the same products for sale. The order of the products in the questionnaire was randomized, and they were offered at only one price level. For products that were also used in experiment 1, that was the middle price level. Results are presented in Table 5.

### Experiment 3

This experiment was set up in the same way as experiment 2, but with some minor alterations. The experiment involved two groups of 250 people, where again each group saw three products. We dropped product 2 because it was shown not to be popular enough in experiment 2, and replaced it by product 6. So the first group of 250 respondents saw products 1, 4, and 6; the other group saw products 5, 6, and 10. The products were offered at the same price levels as in the experiment 2. Results are presented in Table 5.

**Fig 1:**
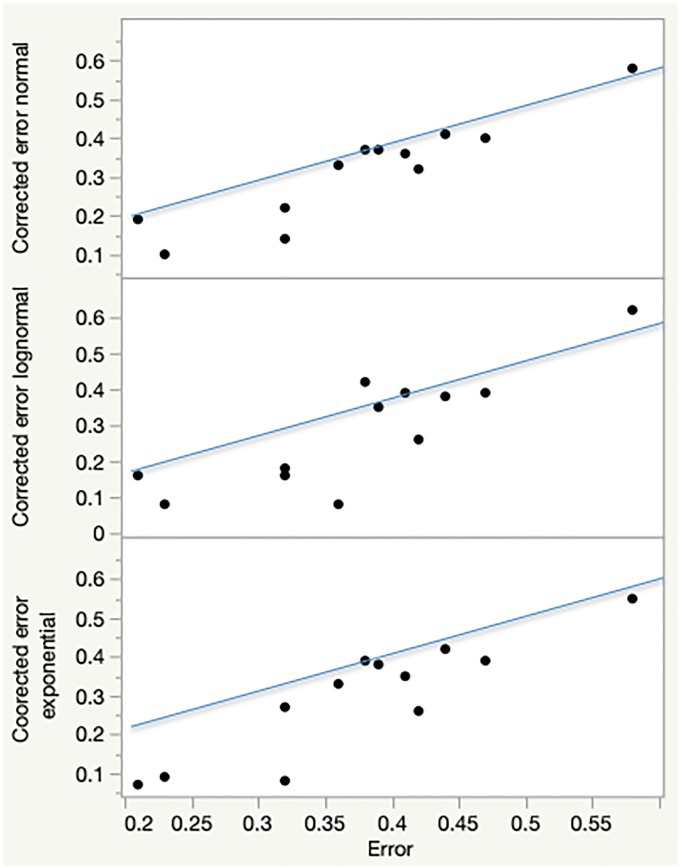
Matched pairs in Experiments 2 and 3.

Matched pairs analysis of the errors in Table 5 yields that the error produced by corrected purchase intentions is significantly smaller compared to the one produced by initial purchase intentions, and that is true for all three models. For illustration, matched pairs for both Experiment 2 and Experiment 3 are graphically presented in Fig 1.

It is noticeable that the real sales in Table 5 are very small. After we observed that fact in experiment 2, we added an open ended question in the experiment 3 where we asked the subjects if anything precluded them in following through with their purchase decisions. In both subject groups, about 20 subjects reported being deterred by the transactional details: namely providing a shipping address and writing a check to the university. Some subjects did not utilize checks any more, and some were worried about the privacy issues. If all of those subjects were not discouraged, the sales in experiment 3 could be expected to rise by about 8%. Even then, if we increase all real sales by 8%, the corrected estimates will be closer to real outcome compared to purchase intentions (one-tailed Wilcoxon tests for all three models are less than p = 0.001).

## Conclusion

Many areas of economics use subjective data, despite the misgivings regarding data quality and reliability. This issue goes beyond truth telling, as subjects may simply lack adequate understanding of the subject matter or fail to pay sufficient attention. In this paper we show how meta-predictions can be used to identify credible respondents, thus improving data reliability. As a test bed for this approach we use purchase intention survey, often used for early sales forecasting of new concepts.

In order to improve data quality and subsequent forecasting precision, we propose to identify those respondents who are of “higher quality” and use only their data while disregarding the information from the others. We address this issue by building a theoretical model based on the predictions score which is used to reward respondents’ meta-knowledge [1], and which can be used to identify people who are best in predicting their peers’ intentions. We show that these best predictors are in fact the credible respondents we seek to detect. We develop an easy algorithm for model application, which allows for correction of purchase intentions by retaining the data from reliable respondents only. It is important to notice that the algorithm requires only one additional question apart from the purchase intention question, which is the meta-prediction question.

The theory is tested in three online experiments. In each experiment, we identify and discard data from unreliable respondents, and base our predictions on only the remaining respondents. The first experiment did not use real purchases, but instead approximated them by the decision to enter a lottery under the understanding that the lottery winner was expected to buy the product. This approach resulted in large purchase intentions, perhaps because subjects treated the decision as almost surely hypothetical (the probability of winning the lottery was small). The other two experiments used real purchases to measure purchase incidence. Those experiments confirmed the hypothesis that corrected purchase intentions produced significantly better predictions of purchase incidence.

Our approach can be generalized to situations where there are more than two possible options and more than two states of the world. We show that regardless of the number of offered answers and possible states of the world, reliable respondents are always those who have the highest prediction scores. In this paper we used purchase intention survey to test the approach, but any survey can be approached in the same way. The only requirement is that some respondents have expertise that allows them to determine with certainty which state of the world has materialized.

A limitation of this paper is that we are not able to directly verify that people whom we identify as credible are really more informed and more attentive than other respondents: instead we do this indirectly by comparing our predictions with the actual outcome. In our present setting, the direct verification would have required additional probing of individual respondents to assess their level of knowledge and attention, something best done through face-to-face interviews, an approach which was not compatible with online panel setting. The direct verification is an issue that we will address in further research via a different set of experiments.

## Supporting information

S1 Appendix (DOCX)
