# Supplementary material for: Whose data can we trust: How meta-predictions can be used to uncover credible respondents in survey data
Source: PLoS One. 2019 Dec 2;14(12):e0225432. doi: 10.1371/journal.pone.0225432 (PMC6886803; doi:10.1371/journal.pone.0225432)
Supplement: S1 Appendix — (DOCX) [file pone.0225432.s001.docx]

APPENDIX

Here we show how the results can be generalized to surveys with more than one option. Assume that the multiple-choice question has m answers. We still assume only two states of nature $\omega^{h}$ and $\omega^{l}$. Then we define respondent types $T_{h1}, T_{h2},\ldots, T_{hm}$ as those who “live” in the state $\omega^{h}$. Type $T_{hi}$ has chosen the answer *i* ; the probability of choosing that answer is $a_{i}$. The respondents who “live” in the state $\omega^{l}$ are of types $T_{l1}, T_{l2},\ldots, T_{lm}$. The probability that type $T_{li}$ chooses answer *i* is $c_{i}$. Unreliable respondents also have m possible choices, so we have m possible types $T_{u1}, T_{u2},\ldots, T_{um}.$They do not recognize the state of the world, so they choose answer *i* with probability $\delta_{i}$in both states of the world. By $\lambda_{i}$ we denote the probability that a reliable respondents chooses answer *i.* Now the matrix of joint probabilities looks like following (please see Table B1):

Table B1. Joint matrix of probabilities

|  |  | $\omega^{h}$ | $\omega^{l}$ |
| --- | --- | --- | --- |
| $T_{h1}$ |  | $\lambda_{1}{a_{1}}p$ | 0 |
| … |  | … | … |
| $T_{hi}$ |  | $\lambda_{i}a_{i}p$ | 0 |
| … |  | … | … |
| $T_{hm}$ |  | $\lambda_{m}a_{m}p$ | 0 |
| $T_{l1}$ |  | 0 | $\lambda_{1}{c_{1}}p$ |
| … |  | … | … |
| $T_{li}$ |  | 0 | $\lambda_{i}c_{i}p$ |
| … |  | … | … |
| $T_{lm}$ |  | 0 | $\lambda_{m}c_{m}p$ |
| $T_{u1}$ |  | ${(1-\lambda}_{1}){\delta_{1}}p$ | ${(1-\lambda}_{1}){\delta_{1}}(1-p)$ |
| … |  | … |  |
| $T_{ui}$ |  | ${(1-\lambda}_{i}){\delta_{i}}p$ | ${(1-\lambda}_{i}){\delta_{i}}(1-p)$ |
| … |  | … | … |
| $T_{um}$ |  | ${(1-\lambda}_{m}){\delta_{m}}p$ | ${(1-\lambda}_{m}){\delta_{m}}(1-p)$ |

From the above matrix we can easily produce the matrix of posteriors, which is as follows (please see Table B2):

Table B2. Matrix of posterior probabilities

|  |  | $\omega^{h}$ | $\omega^{l}$ |
| --- | --- | --- | --- |
| $T_{h1}$ |  | $1$ | 0 |
| … |  | … | … |
| $T_{hi}$ |  | $1$ | 0 |
| … |  | … | … |
| $T_{hm}$ |  | $1$ | 0 |
| $T_{l1}$ |  | 0 | $1$ |
| … |  | … | … |
| $T_{li}$ |  | 0 | $1$ |
| … |  | … | … |
| $T_{lm}$ |  | 0 | $1$ |
| $T_{u1}$ |  | $p$ | $(1-p)$ |
| … |  | … |  |
| $T_{ui}$ |  | $p$ | $(1-p)$ |
| … |  | … | … |
| $T_{um}$ |  | $p$ | $(1-p)$ |

In the same way as before, we can show that the credible respondents in both states of the world are those who have highest prediction scores.

***Proposition B1.***

*Reliable respondents in both states of the world are identical with those who have highest prediction scores.*

Proof :

By “option i” we denote the i-th of the m multiple choice answers.

Assume the state of the world is $\omega^{h}.$First we compute players’ predictions scores as follows:

$P\left( option i | T_{hi} \right)=\alpha_{i}, i=1,\ldots,m$ ; $P\left( option i | T_{ui} \right)=p\alpha_{i}+\left( 1-p \right)\beta_{i}, i=1,\ldots,m.$

Then we compute

$$PS\left( T_{hi} \right)=\sum_{i=1}^{m} \alpha_{i} \cdot log(P\left( option i | T_{1} \right))-\sum_{i=1}^{m} \alpha_{i}log(\alpha_{i})=\sum_{i=1}^{m} \alpha_{i}\cdot log(\alpha_{i})-\sum_{i=1}^{m} \alpha_{i}\cdot log(\alpha_{i})=0.$$

$PS$($T_{ui})=\sum_{i=1}^{m} \alpha_{i}\cdot log(P\left( option i | T_{5} \right)- \sum_{i=1}^{m} \alpha_{i}log(\alpha_{i})=\sum_{i=1}^{m} \alpha_{i}\cdot log(p\alpha_{i}+\left( 1-p \right)\beta_{i})-\sum_{i=1}^{m} \alpha_{i}log(\alpha_{i})$

As $\sum_{i=1}^{m} \alpha_{i}=1$, and $\sum_{i=1}^{m} \beta_{i}=1,$ therefore $\sum_{i=1}^{m} \alpha_{i}p+\left( 1-p \right)\beta_{i}=1$. Now as before we can utilize Gibbs inequality to show that $\sum_{i=1}^{m} \alpha_{i}\cdot\log\left( p\alpha_{i}+\left( 1-p \right)\beta_{i} \right)<\sum_{i=1}^{m} \alpha_{i}log(\alpha_{i})$ which implies that $PS$($T_{ui})<0= P\left( Y | T_{hi} \right)=\alpha_{i}, i=1,\ldots,m$.

In the state of the world $\omega^{l}$ we have $P\left( option i | T_{li} \right)=\beta_{i}, i=1,\ldots,m .$ Similarly as above we show that $PS$($T_{ui})<0= P\left( option i | T_{li} \right)=\beta_{i}, i=1,\ldots,m$.

Q.E.D.

Now we assume arbitrary many states of the world, say *n* states. For a state of the world $\omega^{k},$ which can occur with probability $p_{k},$we introduce a set of credible types $T_{k1,\ldots,}T_{km}$ who “live “ only in the state of the world $\omega^{k}$. The unreliable respondents are denoted by $T_{u1,\ldots,}T_{um}$and they live in all states of the world. The matrix of joint probabilities is a generalization of Table B1, where $P\left( \omega^{k},T_{ki} \right)=$ $\lambda_{i}a_{ki}p_{k},$and $P\left( \omega^{k},T_{ji} \right)=0$ for all other respondent types. For the unreliable types, we have that corresponding joint probabilities are ${P\left( \omega^{k},T_{ui} \right)=(1-\lambda}_{i}){\delta_{i}}p_{k}$. Notice $\sum_{k=1}^{n} p_{k}=1$.

Then we compute the matrix of posteriors. For simplicity we show an example with only three states of the world (please see Table B3).

Table B3. Matrix of posterior probabilities

|  | $\omega^{1}$ | $\omega^{2}$ | $\omega^{3}$ |
| --- | --- | --- | --- |
| $T_{11}$ | $1$ | 0 | 0 |
| … | … | … | … |
| $T_{1i}$ | $1$ | 0 | 0 |
| … | … | … | … |
| $T_{1m}$ | $1$ | 0 | 0 |
| $T_{21}$ | 0 | $1$ | 0 |
| … | … | … | … |
| $T_{2i}$ | 0 | $1$ | 0 |
| … | … | … | … |
| $T_{2m}$ | 0 | $1$ | 0 |
| $T_{31}$ | 0 | 0 | $1$ |
| … | … | … | … |
| $T_{3i}$ | 0 | 0 | $1$ |
| … | … | … | … |
| $T_{3m}$ | 0 | 0 | $1$ |
| $T_{u1}$ | $p_{1}$ | $p_{2}$ | $p_{3}$ |
| … | … | … | … |
| $T_{ui}$ | $p_{1}$ | $p_{2}$ | $p_{3}$ |
| … | … | … | … |
| $T_{um}$ | $p_{1}$ | $p_{2}$ | $p_{3}$ |

For each state of the world $\omega^{k}$we use the proof of proposition B1 to show that the credible respondents $T_{ki}, i=1,\ldots,m$ are the ones with the highest prediction scores.
